# Electroconvulsive Shock Enhances Responsive Motility and Purinergic Currents in Microglia in the Mouse Hippocampus

**DOI:** 10.1523/ENEURO.0056-19.2019

**Published:** 2019-04-23

**Authors:** Alberto Sepulveda-Rodriguez, Pinggan Li, Tahiyana Khan, James D. Ma, Colby A. Carlone, P. Lorenzo Bozzelli, Katherine E. Conant, Patrick A. Forcelli, Stefano Vicini

**Affiliations:** 1 Department of Pharmacology and Physiology, Georgetown University, Washington, DC 20007; 2 Interdisciplinary Program in Neuroscience, Georgetown University, Washington, DC 20007; 3 Department of Neuroscience, Georgetown University, Washington, DC 20007; 4 Department of Pediatrics, Sun Yat-Sen Memorial Hospital, Sun Yat-Sen University, Guangzhou, China

**Keywords:** ATP, ECT, epilepsy, neuroinflammation, patch-clamp electrophysiology, seizures

## Abstract

Microglia are in a privileged position to both affect and be affected by neuroinflammation, neuronal activity and injury, which are all hallmarks of seizures and the epilepsies. Hippocampal microglia become activated after prolonged, damaging seizures known as status epilepticus (SE). However, since SE causes both hyperactivity and injury of neurons, the mechanisms triggering this activation remain unclear, as does the relevance of the microglial activation to the ensuing epileptogenic processes. In this study, we use electroconvulsive shock (ECS) to study the effect of neuronal hyperactivity without neuronal degeneration on mouse hippocampal microglia. Unlike SE, ECS did not alter hippocampal CA1 microglial density, morphology, or baseline motility. In contrast, both ECS and SE produced a similar increase in ATP-directed microglial process motility in acute slices, and similarly upregulated expression of the chemokine C-C motif chemokine ligand 2 (CCL2). Whole-cell patch-clamp recordings of hippocampal CA1*sr* microglia showed that ECS enhanced purinergic currents mediated by P2X7 receptors in the absence of changes in passive properties or voltage-gated currents, or changes in receptor expression. This differs from previously described alterations in intrinsic characteristics which coincided with enhanced purinergic currents following SE. These ECS-induced effects point to a “seizure signature” in hippocampal microglia characterized by altered purinergic signaling. These data demonstrate that ictal activity per se can drive alterations in microglial physiology without neuronal injury. These physiological changes, which up until now have been associated with prolonged and damaging seizures, are of added interest as they may be relevant to electroconvulsive therapy (ECT), which remains a gold-standard treatment for depression.

## Significance Statement

Epilepsy is the fourth most prevalent neurologic disease, affecting one in 26 people over their lifetime. There is a critical unmet need in understanding basic mechanisms underlying the development of epilepsy (epileptogenesis), given that no disease-modifying treatments are currently available. How specific features of microglial activation contribute to subsequent epileptogenesis, and how seizure activity, per se, triggers changes in microglial responses is understudied. In this study, we demonstrate that hippocampal microglia react acutely to single non-epileptogenic seizures, in ways reminiscent of SE-induced activation. Thus, key features of the microglial activation pattern observed after SE may not be related to the epileptogenic process, and further work is needed to fully characterize the interplay between microglia, seizures, and epilepsy.

## Introduction

Neuroinflammation and microglial activation are hallmarks of many neurologic and neurodegenerative diseases, including the epilepsies (Vezzani et al., 2011; Devinsky et al., 2013). As the resident immune macrophage population of the brain, microglia are in a privileged position to monitor neuronal health and activity, and to respond to neuronal injury or hyperactivity (Eyo et al., 2017). Beyond the traditional views of differential reactive microglial states leading to either pro-inflammatory or anti-inflammatory signaling, microglia have distinct and well-established roles in critical components of development (Stevens et al., 2007; Tremblay et al., 2010), physiology (Tremblay et al., 2011; Schafer et al., 2013), and pathology (Hong et al., 2016; Vasek et al., 2016). These roles include synaptic pruning (Stevens et al., 2007) or displacement (Chen et al., 2014), neuronal phagocytosis (Fu et al., 2014) or trogocytosis (Weinhard et al., 2018), and neurotrophic support (Parkhurst et al., 2013).

It is now clear that microglia respond to epileptogenic insults in acquired models of epilepsy (Avignone et al., 2008, 2015; Shapiro et al., 2008; Menteyne et al., 2009; Ulmann et al., 2013; Arisi et al., 2015; Patterson et al., 2015; Rettenbeck et al., 2015; Sabilallah et al., 2016; Wyatt-Johnson et al., 2017; Kalozoumi et al., 2018; Klement et al., 2018). Furthermore, a recent study suggests that noninflammatory microglial changes could be the main driver of epileptogenesis in a mouse model of tuberous sclerosis (Zhao et al., 2018), although the specificity of the genetic tools used to target microglia in that study has been debated (Zou et al., 2017; Zhang et al., 2018).

Much like other epileptogenic insults to the brain (Klein et al., 2017), status epilepticus (SE) induces a wide spectrum of changes in pro-inflammatory and anti-inflammatory cytokine expression and is accompanied by microglial proliferation and morphologic/physiologic activation (Avignone et al., 2008; Menteyne et al., 2009). Notably, SE triggers enhanced purinergic signaling in microglia, which correlates with increased velocity of microglial process motility (Avignone et al., 2008, 2015; Ulmann et al., 2013). It is tempting to speculate that this response is an active contributor to the epileptogenic process. However, little is known about the effect of acute seizures/non-epileptogenic hyperactivity on these cells, making it imprudent to assume that they represent putative antiepileptogenic targets.

Moreover, because experimental SE in rodents also acutely causes extensive hippocampal degeneration (Turski et al., 1984; Pollard et al., 1994; Avignone et al., 2008) and blood-brain barrier breakdown (Marchi et al., 2007; van Vliet et al., 2007; Gorter et al., 2015), it is still unclear how much of the post-SE microglial activation is a result of the seizure activity per se, as opposed to the brain damage downstream of the epileptic crisis: it is plausible that the SE-induced injury (and not the SE itself) is the chief contributor to the particular SE-induced microglial activation.

To isolate the role of acute seizures from that of SE-induced damage, we used electroconvulsive shock (ECS), an acute seizure model that is not associated with epileptogenesis nor neuronal cell death (Orzi et al., 1990; Devanand et al., 1994; Scott, 1995; Conti et al., 2009; Basar et al., 2013; van Buel et al., 2017). Repeated ECS in rodents models human electroconvulsive therapy (ECT) with near-perfect validity (Li et al., 2007; van Buel et al., 2017). Above and beyond the established utility of ECS in the preclinical screening of anti-seizure drugs, ECT is the most reliable and effective treatment for major depressive disorder (MDD) available in the clinic today (UK ECT Review Group, 2003; Weiner and Reti, 2017). We found that hippocampal microglia responded to single ECS seizures with a striking upregulation of purinergic signaling and responsive process motility. Our new data are thus positioned to both address the role of acute hyperactivity-induced microglial activation in epilepsy, and also begin to unravel if hippocampal microglia participate in the mode of action of a best-in-class therapy against depression.

## Materials and Methods

### Experimental animals

We used postnatal day (P)30–P45 male and female CX_3_CR1^GFP/+^ mice, which possess green fluorescent protein in place of one allele of the fractalkine receptor gene, resulting in fluorescently labeled microglia (Jung et al., 2000). CX_3_CR1^GFP/GFP^ mice (005582, RRID:IMSR_JAX:005582) were obtained from The Jackson Laboratory and backcrossed onto a C57BL/6J (000664, RRID:IMSR_JAX:000664) congenic background before this study. A total of 143 mice were used for the experiments in this study. All animals used in this study were housed on a standard 12/12 h light/dark cycle with water and standard chow available *ad libitum*.

Experimental and control mice were heterozygous littermates resulting from homozygote × wild-type mating. Littermates were randomized to treatment and control groups. Matched control animals were handled and treated in the same manner except that they received sham/0 mA shocks or saline/vehicle instead of pilocarpine injections (see below). Vehicle and sham control animals were pooled in a single control group for all analyses. The procedures described in this manuscript were performed with the approval of the Georgetown University Animal Care and Use Committee.

### Maximal electroshock

Maximal (tonic-clonic) seizures were elicited by transcorneal stimulation using an Ugo Basile Electro-Convulsive Device (57800, Stoelting Co.) and custom-built transcorneal electrodes. Animals received 0.5% tetracaine HCl eyedrops (Alcon) 15–30 min before stimulation. Shocks consisting of 0.3 s long trains of 0.9 ms wide square pulses (17 mA for females, 19 mA for males) at 200 Hz reliably evoked a tonic hindlimb extension response lasting between 12 and 20 s with negligible mortality. Stimulation protocols and intensities were adapted from the literature (Frankel et al., 2001) and the associated Mouse Phenome Database (The Jackson Laboratory) dataset “Frankel1” publicly available online at https://phenome.jax.org/projects/Frankel1/protocol.

### SE

For the SE model (all injections were intraperitoneal, and all drugs were dissolved in sterile 0.9% NaCl unless noted otherwise): 30 min after pretreating with 1 mg/kg each of scopolamine methylbromide and terbutaline (Cho et al., 2015), we injected 260 to 320 mg/kg pilocarpine HCl, and observed the animals as they progressed through modified Racine stages (1 = mouth and face automatisms, 2 = head bobbing, 3 = unilateral forelimb clonus, 4 = bilateral forelimb clonus, and 5 = rearing and falling; Racine, 1972) and into SE (defined as continuous seizures over stage 3 for longer than 5 min). SE was terminated after 2 h by injecting diazepam (10 mg/kg). Concurrently, mice received 0.25 ml (s.c.) of sterile warmed dextrose acetate Ringer’s solution. Ethosuximide (150 mg/kg, s.c., in PBS) was administered 6 h after the start of seizures, together with 0.3 ml of sterile 0.9% NaCl (Pearce et al., 2014; Iyengar et al., 2015). During and immediately after SE, mice were kept huddled/touching in a bare warmed (30–31°C) cage. This induction protocol reliably elicited SE in all our mice, with limited mortality (3/21 mice died during the seizures).

### Acute slice preparation

Twenty-four hours following seizure induction, mice were euthanized by decapitation and brains were rapidly dissected into an ice-cold sucrose aCSF slicing solution as previously described (Al‐Muhtasib et al., 2018), and in a manner consistent with the AVMA Guidelines on Euthanasia of Laboratory Animals (American Veterinary Medical Association, 2013). The slicing solution contained: 88 mmol/l NaCl, 2.7 mmol/l KCl, 0.5 mmol/l CaCl_2_, 6.6 mmol/l MgSO_4_ anhydrate, 1.4 mmol/l NaH_2_PO_4_, 26 mmol/l NaHCO_3_, 25 mmol/l dextrose, and 75 mmol/l sucrose (all chemicals from Sigma, unless otherwise noted); 300 μm horizontal hippocampal slices were cut on a Vibratome 3000 Plus Sectioning System (Vibratome), in cold sucrose aCSF as above. Sections were recovered for 30 min at 32°C in normal/recording aCSF solution, containing: 124 mmol/l NaCl, 4.5 mmol/l KCl, 1.2 mmol/l Na_2_HPO_4_, 26 mmol/l NaHCO_3_, 2 mmol/l CaCl_2_, 1 mmol/l MgCl_2_, and 10 mmol/l dextrose. Slices were then transferred to room temperature (RT; 22–24°C) and equilibrated for >10 min before use. In some experiments, slices were incubated with 1.8 μM sulforhodamine 101 in aCSF for 10 min to mark astrocytes (Nimmerjahn et al., 2004) for unrelated studies (data not shown). To limit artifactual microglial activation from dissection/sectioning, all slices were used within 5 h of euthanasia, and all microglia studied had somata at least 10 μm away from the cut surfaces. aCSF solutions were maintained at pH 7.4 by bubbling with carbogen gas (95% O_2_/5% CO_2_, Roberts Oxygen). All experiments were conducted at RT.

### Patch-clamp electrophysiology

Whole-cell patch-clamp recordings were performed under DIC illumination on fluorescently identified ramified cells, with 4.5 to 6.5 MΩ pipettes pulled from Wiretrol II borosilicate glass capillaries (Drummond) filled with an internal solution as described in Avignone et al. (2008), containing: 132 mmol/l K-gluconate, 11 mmol/l HEPES, 0.1 mmol/l EGTA, and 4 mmol/l MgCl_2_; pH 7.35 adjusted with KOH.

Liquid junction potential (LJP; −14 mV) was calculated in Clampex11 (pClamp, RRID:SCR_011323, Molecular Devices). The LJP correction was only applied to our reported resting membrane potential (RMP) values. Patch-clamp was performed with a MultiClamp700B amplifier (Molecular Devices). Recordings were digitized at 20 kHz and low-pass Bessel-filtered at 2 kHz with a personal computer running Clampex11 and a DigiData1440 (Molecular Devices).

RMP was measured in current clamp (I = 0) mode, immediately after break-in to minimize effect of dialysis. All other data were recorded in voltage clamp configuration. Input resistance (IR), access resistance and cell capacitance were calculated from the current response to brief −5 mV hyperpolarizing voltage steps. I/V curves were calculated using 500-ms voltage steps from −60 mV to Vm = −120 mV to +50 mV, every 10 mV.

For agonist-evoked current studies, cells were held at Vm = −60 mV, with 500 ms voltage ramps from −120 to −50 mV delivered every 10 s; 1 mM Na-ATP in normal or divalent cation-free/0CaMg aCSF (aCSF as described above, minus CaCl_2_ and MgCl_2_) was locally perfused via a custom-made Y-tube apparatus (Murase et al., 1989; Hevers and Lüddens, 2002). Recordings were analyzed offline with Clampfit 10.7 and 11.3 (pClamp, Molecular Devices). Results are shown as current density (C.D., in pA/pF) to take varying cell sizes/capacitances into account. Access resistance was monitored periodically during the experiment and recordings with change >20% were discarded.

For the P2X7 current studies, Brilliant Blue G (BBG; B0770, Sigma) was dissolved in aCSF to create a 1 mM stock solution, and then some slices were preincubated in 5 μM BBG in aCSF for 30 min (Avignone et al., 2008). Electrophysiology recordings were then conducted as outlined above but in the presence of 5 μM BBG.

### Confocal imaging

Confocal Z- or ZT-stacks were taken using a laser scanning microscope system (Thor Imaging Systems Division) equipped with 488/561/642 nm lasers and Green/Red/Far-red filters and dichroics and mounted on an upright Eclipse FN1 microscope (Nikon Instruments); 284 × 284 × 20 μm (*xyz*) volumes of horizontal hippocampal slices containing CA1*sr* were imaged with a long working distance 60× water-dipping objective (CFI Fluor 60XW, NA = 1.0, WD = 2 mm, Nikon). Differential interference contrast (DIC) images (on acute and fixed slices) or fluorescent images of NeuN (for neuronal nuclei) staining (on fixed slices only, see below) were used to identify and confirm our region of interest as CA1*sr*.

### Microglial motility

For the baseline and responsive motility experiments, we took 1024 × 1024 pixel (px) ZT-stacks of acute slices by imaging 11 planes 2 μm apart every 30 s. If necessary, maximal intensity projection (MIP over the *z*-axis) time lapses were registered using the StackReg plugin (Thevenaz et al., 1998) in FIJI (RRID:SCR_002285, Wayne Rasband/NIH) before the motility analyses.

### Baseline motility

Spontaneous motility of microglial processes directly mediates the physical microglia-neuron contact that is a prerequisite for many microglial functions like phagocytosis of synaptic terminals. We imaged this baseline behavior over 20 min in CA1*sr* of either naïve slices or in the presence of a 0 mM [ATP] containing (aCSF-only) pipette (controls for responsive motility, see below).

Motility analysis was performed in FIJI by adapting the method described in Eyo et al. (2018). We first manually cropped, then automatically thresholded and binarized the ROIs. The area above threshold at the end of the time-lapse movie (*t* = 20 min) was then measured and normalized to the area above threshold of the first frame of the movie [*t* = 0, extension index (EI) = 1.0]. The EI through time of each time-lapse movie was then determined.

### Responsive motility

Responsive motility of microglial processes is a vital endogenous response to injury (Davalos et al., 2005), and is a sensitive and reproducible in-slice assay of microglial purinergic signaling. In an assay adapted from the work of Avignone et al. (2008), we lowered a patch pipette containing 1, 3, or 10 mM [Na-ATP] in aCSF into CA1*sr*, 10–20 μm deep, and imaged the volume surrounding it for 20 min. We quantified process velocity with the Manual Tracking plugin in FIJI (Cordelieres, 2018). Between three and eight responding processes per slice were manually tracked as they moved toward the pipette. Control experiments with 0 mM ATP (aCSF only in the pipette) did not elicit any appreciable directional motility in the processes of nearby microglia.

### Tissue sectioning

Animals were anesthetized with unmetered isoflurane (Patterson Veterinary) or pentobarbital (>100 mg/kg) and intracardially perfused with cold PBS. Brains were quickly excised and drop-fixed overnight in 4% paraformaldehyde (18505, Ted Pella Inc.) + 4% sucrose in PBS; 50 to 100 μm hippocampal slices were cut horizontally using a Vibratome Series 1000 (Vibratome) for immunostaining and morphometry. For FluoroJade C (FJC) studies, fixed brains were cryoprotected overnight in 30% sucrose in PBS before freezing, and sectioned at 25 μm on a cryostat (CM1850, Leica Biosystems) and immediately mounted on 10–12 gelatin-subbed slides per brain.

### Microglial density

For microglial density quantification we took 2048 × 2048 px Z-stacks of fixed slices from perfused brains by imaging 21 planes 1 μm apart. We analyzed the MIPs across the Z axis, referring to the 3D Z-stack for the manual cell counting analysis if necessary. Cells were manually counted using FIJI in a single 284 × 284 × 20 μm field containing CA1*sr* per hemi section.

### Microglial morphology

Following perfusion and sectioning, slices were processed free-floating for immunofluorescence against GFP to better visualize microglia and their fine processes, and against NeuN to mark stratum pyramidale. Sections were blocked and permeabilized for 2 h in 0.5% Triton X-100 and 10% normal goat serum in PBS. Next, slices were incubated overnight at 4°C with mouse anti-GFP (1:1000, Millipore Bioscience Research Reagents MAB3850, RRID:AB_94936, MilliporeSigma) and rabbit anti-NeuN (1:500, ABN78, RRID:AB_10807945, MilliporeSigma). Slices were washed and then incubated at RT for 1 h with secondary antibodies (1:1000 each; goat anti-mouse AlexaFluor647, A-21235, RRID:AB_2535804, Thermo Fisher Scientific; goat anti-rabbit Cy3, 111-165-144, RRID:AB_2338006, Jackson ImmunoResearch). Sections were mounted and coverslipped using VectaShield fluorescent mounting media (H-1200, RRID:AB_2336790, Vector Laboratories).

Individual microglia were traced using the “FilamentTracer” tool in Imaris 7.4.2 (RRID:SCR_007366, Bitplane) from Z-stacks of fixed anti-GFP stained slices with 41 planes of 4096 × 4096 px taken at 0.5 μm apart. We compared microglia morphometrically by extracting patterns of 3D Sholl crossings, numbers of branching points and primary branches, and total filament tree lengths for each traced cell.

### FJC staining

To visualize neuronal damage, we used FJC, a polyanionic fluorescein derivative that can selectively mark degenerating neurons (Schmued et al., 2005). We employed an FJC Ready-to-Dilute kit (TR-100-FJC, Biosensis) and followed the manufacturer’s instructions, except for halving the time in potassium permanganate.

Briefly, after drying, slides were treated with basic ethanol solution for 5 min before transfer into 70% ethanol for 2 min, then rinsed in distilled/deionized water (ddH_2_O) for 2 min. After incubating in a 0.06% potassium permanganate solution for 5 min, followed by a 2 min rinse in ddH_2_O, samples were stained in an acidified 0.001% FJC working solution for 10 min in the dark. After staining, slides were washed three times for 1 min in ddH_2_O, then placed on a slide warmer at 40°C until dry before being cleared in xylene for 2 min and coverslipped with D.P.X. mounting medium (13510, Electron Microscopy Sciences). Fluorescence photomicrographs from three to five sections per slide were captured on an upright microscope (i80, Nikon Instruments) with a QIClick camera (QImaging), using a standard FITC filter set and a 0.65NA 40× objective (Nikon Instruments). Images were captured by a blinded investigator using the same imaging conditions throughout. FJC-positive cells in each image were manually counted by two blinded investigators. Cell counts were averaged from at least three sections per animal.

### Microglial isolation

Microglial isolation was performed 24 h after ECS seizures, exploiting the magnetic activated cell sorting (MACS) approach with anti-Cd11b MicroBeads (130-049-601, all MACS supplies are from Miltenyi Biotec), tightly adhering to the manufacturer’s standard protocol for single cell dissociation and microglial isolation from adult brains, except for omission of the erythrocyte lysis step.

Mice were anesthetized and perfused transcardially with cold PBS. Brains were rapidly dissected on ice and stored in MACS tissue storage solution (130-100-008) until dissociation could proceed (<15 min). Brain tissues were sliced six to eight times with a sterile scalpel, then placed together with the enzyme mix from the adult brain dissociation kit (130-107-677) in a C tube (130-093-237) for processing in the gentleMACS Octo Dissociator with Heaters (130-096-427) using the recommended program (37C_ABDK_01). The dissociated tissue was resuspended in PBS and applied to a 30 µm SmartStrainer (130-098-458). All subsequent steps were performed at 4°C except for the magnetic column separation. The resulting single-cell suspension was centrifuged at 300 × *g* for 10 min at 4°C (Allegra X-30R, Beckman Coulter), and the supernatant aspirated. The cell pellet was resuspended in 3100 µl of PBS and mixed with 900 µl of debris removal solution; 4 ml of cold PBS was then overlaid on top, and the tubes were centrifuged for 10 min at 3000 × *g*. Three phases formed, the top two were discarded and cold PBS was added to the tube to bring the final volume to 15 ml, before being centrifuged at 1000 × *g* for 10 min and aspirated completely. The pellet was resuspended in 10 ml cold 0.5% BSA in PBS, and centrifuged at 300 × *g* for 10 min. The supernatant was again aspirated completely, and the cell pellet was carefully resuspended in 90 µl cold 0.5% BSA, to which 10 µl of MicroBeads were added before incubation in the dark for 15 min. The cells were washed in cold 0.5% BSA and centrifuged at 300 × *g* for 5 min, supernatant was aspirated completely, and the pellet was resuspended in 500 µl 0.5% BSA. Microglia were magnetically isolated through MACS MS columns (130-042-201) placed into MiniMACS Separator magnets (130-042-102). Unlabeled cells were collected in the original flow-through and after 3 × 500 µl washes. Labeled microglial cells were collected by flushing the column after removal from the magnetic field.

### RNA extraction and purification

After MACS isolation, positive fractions were immediately lysed with 0.8 ml cold TRIzol LS (Invitrogen 10296028, ThermoFisher Scientific) and homogenized by thorough pipette and vortex mixing. After a 5 min initial incubation, 0.2 ml chloroform (C7559, Sigma) was added and samples were incubated for 2–3 min before being centrifuged for 15 min at 12,000 × *g* and 4°C. The aqueous phase was transferred to a new tube with 0.5 ml isopropanol (I9516, Sigma) and RNA was precipitated by incubating for 10 min at RT. After being centrifuged for 10 min at 12,000 × *g* and 4°C, the supernatant was discarded, and the RNA washed twice by resuspending the pellet in 75% ethanol (E7023, Sigma) in UltraPure Nuclease-free ddH_2_O (Invitrogen 10977023, ThermoFisher Scientific), then vortexing briefly and then spinning down for 5 min at 7,500 × *g* and 4°C. After the second wash was removed, RNA was air-dried for 10 min before resuspension in 25 μl RNase-free water. Samples were then incubated at 60°C for 10–15 min before RNA quantity and purity were determined by standard spectrophotometry methods (NanoDrop 1000, ThermoFisher Scientific). If necessary, RNA samples were stored at –80°C before cDNA synthesis and qPCR analysis downstream.

### mRNA expression analysis by two-step RT-qPCR

cDNA was synthesized from RNA samples using random hexamer primers in the SuperScript IV First Strand Synthesis system (Invitrogen 18091050, ThermoFisher Scientific) according to the manufacturer’s directions. cDNA samples were stored at –20°C before qPCR analysis; 10% of the 20 μl RT reaction output was used in a 20 μl qPCR reaction for each technical replicate.

Hydrolysis probe-based qPCR reactions were run in multiplex on a 4-channel (FAM, HEX, TEX615, Cy5) Mic cycler instrument (Bio Molecular Systems) using mouse assays from the PrimePCR line (Bio-Rad) as follows (with format *Gene*: Unique AssayID): *Actb*: qMmuCEP0039589; *Tmem119*: qMmuCEP0042925; *Tnf*: qMmuCEP0028054; *Ccl2*: qMmuCEP0056726; *P2rx1*: qMmuCIP0031612; *P2rx4*: qMmuCIP0028782; *P2rx7*: qMmuCIP0042331; *P2ry6*: qMmuCIP0029813; *P2ry12*: qMmuCEP0057087.

PCR reactions were prepared in duplicate or triplicate with Bioline SensiFast Probe No-ROX mastermix (Thomas Scientific) following the manufacturer’s directions for multiplex assays (polymerase activation for 3 min at 95°C; then 40 cycles of 10 s denaturation at 95°C, 50 s annealing/extension at 60°C, signal acquisition). Multiplex assay combinations were validated by comparing their results to those from parallel singleplex reactions (data not shown). Threshold cycle was automatically determined and averaged across replicates by the cycler manager software (Bio Molecular Sciences). Fold changes were determined using the 2^–ΔΔCt^ method, with expression of all transcripts normalized to *Actb* levels in the control group.

### C-C motif chemokine ligand 2 (CCL2) ELISA and total protein quantification on hippocampal lysates

After decapitation, hippocampi were dissected in ice-cold PBS, flash frozen in isopentane cooled in dry ice (−78°C), and lysed (200 μl of RIPA buffer + HALT inhibitor cocktail/hemi-hippocampus, both from ThermoFisher Scientific). Lysates were probed for total protein using a Pierce BCA assay (23227, ThermoFisher Scientific) and for CCL2/monocyte chemoattractant protein 1 (MCP-1) using a Quantikine CCL2 ELISA (MJE00, R&D Systems) assay according to manufacturers’ directions.

### Experimental design and statistical analysis

Our primary question of interest was the degree to which ECS would alter microglial responses. The impact of SE on the same endpoints we measure for ECS have been previously reported and replicated by others (Avignone et al., 2008, 2015; Menteyne et al., 2009; Eyo et al., 2014, 2016a; Arisi et al., 2015; Schartz et al., 2016; Tian et al., 2017; Wyatt-Johnson et al., 2017). For comparison purposes, we included a SE group as a positive control in subset of experiments. We focused our analysis on CA1, as the microglial response to SE has been best characterized in this region. All data were analyzed by investigators blinded to treatment status using FIJI, Clampfit 10.7 and 11, Excel (RRID:SCR_016137, Microsoft) and Prism (RRID:SCR_002798, GraphPad). Results are presented as mean ± SEM. *N* is number of animals and n is number of slices/fields or number of cells for the patch-clamp electrophysiology studies. We did not detect any sex differences in microglial density, morphology and motility, or in microglial gene expression, and thus combined data across sexes for further statistical analysis. Similarly, wherever applicable, control groups for both seizure models (saline vehicle for SE, sham shock for ECS) were combined.

Data were statistically analyzed by two-way ANOVA followed by Sidak’s multiple comparisons test [microglial 3D Sholl analysis; qPCR data], by one-way ANOVA followed by *post hoc* multiple comparisons with Tukey’s test [other microglial morphometry data; CCL2 ELISA analysis data], by Kruskal–Wallis non-parametric test followed by multiple comparisons with Dunn’s correction (for the non-normally distributed BBG preincubation electrophysiology data), or by unpaired *t* tests for all other comparisons. We established statistical significance at *p* < 0.05, applied recommended multiple comparison corrections where appropriate and computed all *p* values from two-tailed distributions. Exact *p* values are provided whenever made available by the statistics software (Prism).

## Results

To characterize the microglial response to neuronal hyperactivity/seizures we employed ECS to induce maximal seizures on CX_3_CR1^GFP/+^ mice and studied green fluorescent protein-labeled microglia in hippocampal area CA1*sr*.

**Figure 1.**
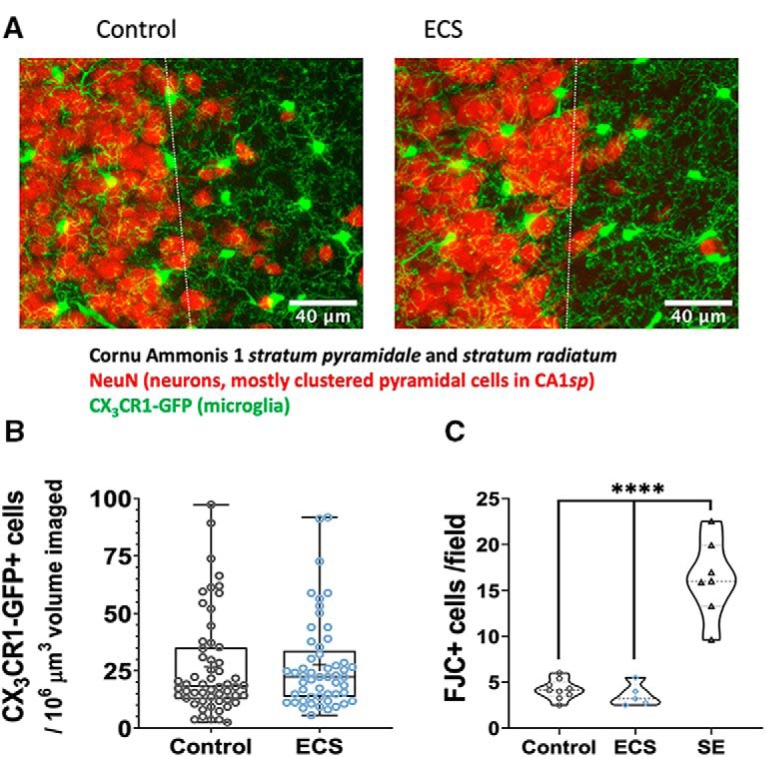
ECS did not affect density of microglia and did not cause neuronal degeneration in mouse CA1 24 h after seizures. ***A***, Representative MIPs from immunofluorescence image showing CX_3_CR1-GFP^+^ microglia in green and NeuN^+^ neurons in red in CA1 (stratum pyramidale = left of dashed line; stratum radiatum = right of dashed line) of control (top) and ECS (bottom) mice 24 h after seizures. ***B***, Density of microglia in CA1*sr* microglia per 10^6^ μm^3^ of CA1*sr* volume. Control animals had 26.69 ± 2.9 which was not significantly different from ECS animals that had 27.59 ± 2.7. ***C***, Number of FJC-positive cells per field imaged (320 × 240 μm). Control and ECS-exposed animals have significantly lower degenerating cell densities in CA1 than SE-exposed animals: 4.2 ± 0.36 for controls (*N* = 9) and 3.6 ± 0.54 for ECS animals (*N* = 5), compared to 16.3 ± 1.5 for SE animals (*N* = 7). ****p < 0.0001.

A single ECS-induced tonic-clonic seizure did not result in observable differences in microglial density or baseline motility 24 h after the seizures in the CA1sr hippocampal region. As shown in the examples in Figure 1*A* and the summary data in Figure 1*B* (by slice/field), CA1 slices from control animals (*N* = 13 mice, *n* = 56 fields) had a mean cell density of 27 ± 2.9 microglia/10^6^ μm^3^ of volume imaged, compared to 28 ± 2.7 in the ECS group (*N* = 12, *n* = 53; by slice: *t*_(107)_ = 0.949, *p* = 0.34, *t* test; by animal: *t*_(23)_ = 0.367, *p* = 0.717, *t* test; data not shown).

To verify that our ECS treatment was not associated with neuronal damage in the hippocampus, we counted FJC-positive cells. FJC is a polyanionic dye that selectively marks degenerating neurons (Schmued et al., 2005). The number of FJC+ cells per 320 × 240 μm field imaged was low in controls (4.2 ± 0.36; *N* = 9) and ECS-exposed animals (3.6 ± 0.54; *N* = 5), but significantly elevated in animals that underwent SE (16.3 ± 1.5; *N* = 7; Fig. 1*C*). Analysis by one-way ANOVA revealed a statistically significant effect of treatment (*F*_(2,18)_ = 53.1, *p* = 0.00000003), that was driven by the SE group, which differed from both control (*q*_(18)_ = 13.13, *p* = 0.00000008, Tukey’s test) and ECS groups (*q*_(18)_ = 11.89, *p* = 0.0000003, Tukey’s test). Control and ECS groups did not differ from each other (*q*_(18)_ = 0.612, *p* = 0.902, Tukey’s test). Thus, as expected, single ECS did not cause acute neurodegeneration, a profile different from that following SE, which is associated with high levels of degeneration.

**Figure 2.**
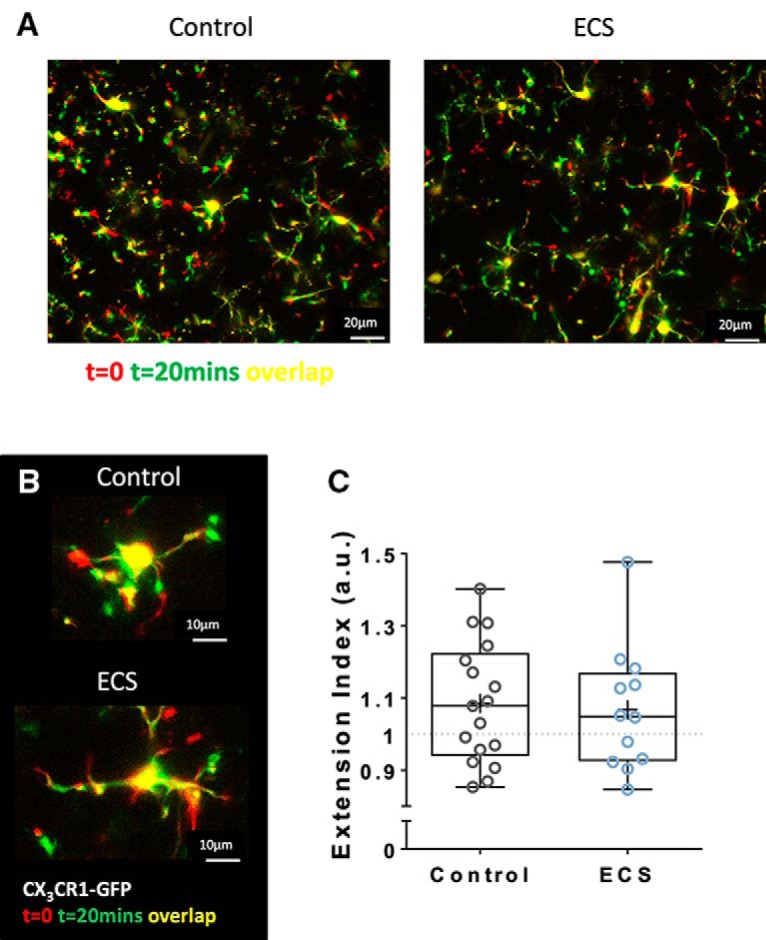
ECS did not affect spontaneous motility of microglia in mouse CA1*sr* 24 h after seizures. ***A***, ***B***, Representative time-coded images (*t* = 0 in red, *t* = 20 min in green, overlap in yellow) of CA1*sr* fields (***A***) or single cells (***B***) from slices from control (right/top) and ECS (left/bottom) treated animals. ***C***, ECS had no effect on mean EI (area of extensions/area of retractions) after 20 min of imaging under baseline conditions: 1.085 ± 0.041 for control slices and 1.067 ± 0.05 for ECS slices.

To evaluate baseline motility, we calculated an EI by dividing the mean area of process extensions by the mean area of retracted processes (Eyo et al., 2018) over an imaged field as shown in the examples in Figure 2*A*,*B*. Over 20 min of imaging under baseline conditions, we measured similar mean extension indices of 1.09 ± 0.04 for control slices and 1.07 ± 0.05 for ECS slices (*N* = 7 and 6 animals; *n* = 17 and 12 slices; *t*_(27)_ = 0.269, *p* = 0.79, *t* test; Fig. 2*C*).

**Figure 3.**
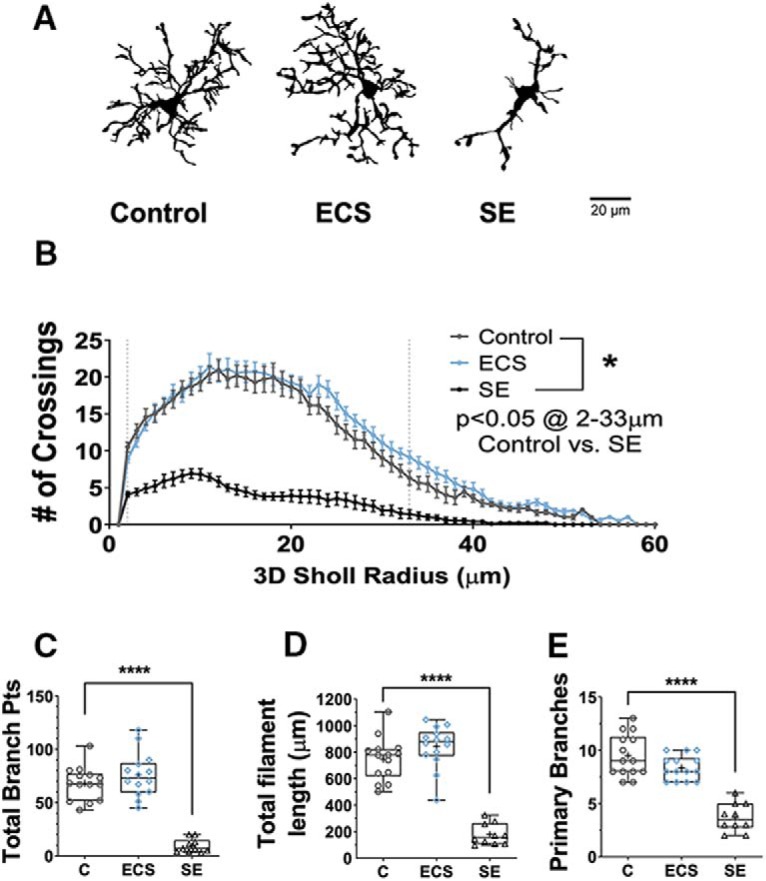
Unlike SE, ECS did not cause morphologic activation of CA1*sr* microglia. ***A***, Representative binarized images of individual traced microglia from control, ECS or SE animals. ***B***, SE, but not ECS, significantly decreased the number of 3D Sholl crossings in hippocampal microglia (for radii between 2 and 33 μm). ***C***, SE significantly decreased the total number of branch points per cell, while ECS had no significant effect. ***D***, SE significantly decreased the total filament length per cell, while ECS had no significant effect. ***E***, SE significantly decreased the average number of primary branches, while ECS had no significant effect. *p < 0.05, ****p < 0.0001.

To investigate the effect of our experimental treatment on microglial morphology we reconstructed confocal z-stacks of individual cells in CA1*sr* after perfusion, fixation, and immunofluorescent amplification of GFP. Representative tracings are shown in Figure 3*A*. We traced a total of 14 cells from seven control animals, 14 cells from seven ECS animals, and 10 cells from five SE animals.

Microglia from control animals were highly ramified and had long and complex processes with regular branching, as is expected under physiologic conditions. Compared to controls, and unlike ECS animals, SE animals displayed clear morphologic activation of hippocampal microglia, as has been previously described by others (Avignone et al., 2008, 2015; Wyatt-Johnson et al., 2017). The 3D Sholl profile was more compact in slices from SE-exposed animals as compared to slices from controls (*F*_(1,1320)_ = 1761, *p* < 10^−15^), with significantly fewer crossings from 2 to 33 μm from the cell body (all *p* < 0.0001, Tukey’s multiple comparisons tests; Fig. 3*B*). Cells from SE-exposed animals had a significantly shorter total process length (748 ± 44 μm in control vs 178 ± 26 μm in SE, *q*_(35)_ = 13.4, *p* = 0.0000000001, Tukey’s test; Fig. 3*C*), and also had significantly fewer branching points (68 ± 4.2 in control vs 9.4 ± 2.0 in SE, *q*_(35)_ = 12.4, *p* = 0.0000000007, Tukey’s test; Fig. 3*D*) and primary branches (9.5 ± 0.53 in control vs 3.7 ± 0.42 in SE, *q*_(35)_ = 12.73, *p* = 0.0000000004, Tukey’s test; Fig. 3*E*). On the other hand, microglia from the hippocampus of ECS-exposed animals had no detectable differences in morphology when compared to controls. Their 3D Sholl profile was similar to control cells (*p* > 0.9 at all radii, Tukey’s multiple comparisons tests; Fig. 3*B*), and they also had comparable total process length (843 ± 43 μm, *q*_(35)_ = 2.422, *p* = 0.22, Tukey’s test; Fig. 3*C*), and number of branching points (76 ± 5.5, *q*_(35)_ = 1.79, *p* = 0.43, Tukey’s test; Fig. 3*D*) and primary branches (8.36 ± 0.31, *q*_(35)_ = 2.747, *p* = 0.142, Tukey’s test; Fig. 3*E*).

Thus, ECS did not induce any observable microglial activation as measured by microglial density, spontaneous motility or morphology. Given that “activated” microglia are at once a result, target, and source of proinflammatory molecules, we wanted to verify that our model was associated with low relative expression levels of CCL2 (also known as MCP-1), a chemokine whose expression was significantly upregulated after SE (Avignone et al., 2008; Foresti et al., 2009; Arisi et al., 2015). CCL2 has been mechanistically implicated in the neuronal cell death that follows SE (Tian et al., 2017).

**Figure 4.**
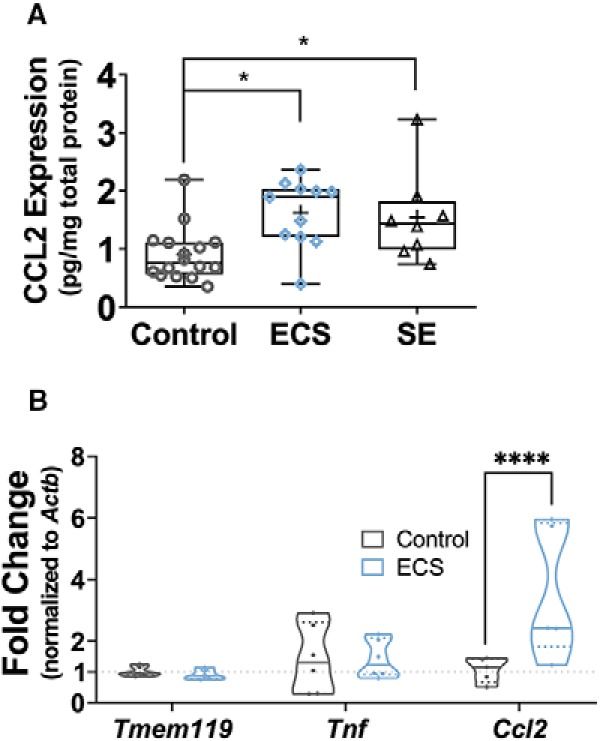
ECS increased expression of CCL2 without affecting *Tnf* or the microglial marker *Tmem119*. ***A***, Hippocampi were lysed 24 h after seizures, and the ratio of CCL2 to total protein was measured in the lysates by ELISA and BCA assay. ECS and SE similarly induced a significant upregulation of relative CCL2 expression. ***B***, After MACS isolation 24 h after ECS or sham ECS, microglial RNA samples were studied by hydrolysis probe-based qPCR. Relative fold change for each transcript assayed (the microglial marker *Tmem119*, the pro-inflammatory cytokine *Tnf* and the chemokine *Ccl2*) was determined by the 2^–ΔΔCt^ method, normalizing to *Actb* levels. We observed significantly higher relative expression of *Ccl2* mRNA in ECS microglial samples and no significant changes elsewhere. *p < 0.05, ****p < 0.0001.

Surprisingly, as shown in Figure 4*A*, ECS and SE induced a similar increase in CCL2 protein levels as measured by ELISA in hippocampal lysates taken 24 h after the seizures. As expected, lysates from control animals displayed low levels of CCL2 (0.9 ± 0.12-pg CCL2/mg total protein; *N* = 16 hippocampi from 16 animals). One-way ANOVA revealed a statistically significant treatment group effect (*F*_(2,32)_ = 6.13, *p* = 0.0056). This effect was driven by a significant increase in CCL2 in the ECS (1.6 ± 0.18-pg/mg total protein, *N* = 11, *q*_(32)_ = 4.48, *p* = 0.0091) and SE (1.5 ± 0.27-pg/mg total protein, *N* = 8, *q*_(32)_ = 3.61, *p* = 0.041) groups, as compared to the control group by Tukey’s test. There was no significant difference in CCL2 protein expression between the ECS and the SE groups (*q*_(32)_ = 0.416, *p* = 0.95). When microglial RNA samples were analyzed by qPCR, two-way repeated measures mixed model ANOVA analysis showed significant effects of treatment group (*F*_(1,28)_ = 5.311, *p* = 0.03), interaction between treatment group and gene (*F*_(2,28)_ = 5.815, *p* = 0.008) as well as of gene assayed (*F*_(2,28)_ = 5.156, *p* = 0.01). As shown in Figure 4*B*, we found *Ccl2* to be significantly changed: in accordance with the increased CCL2 protein expression in ECS hippocampi (Fig. 4*A*), *Ccl2* mRNA levels were significantly upregulated in ECS microglia compared to controls (control FC = 1.07 ± 0.18, ECS FC = 3.56 ± 0.97, *t*_(28)_ = 3.959, *p* = 0.001, *N* = 5/group). No differences were found in relative expression of the microglial marker *Tmem119* (control FC = 1.01 ± 0.07, ECS FC = 0.91 ± 0.07, *t*_(28)_ = 0.175, *p* > 0.99, *N* = 6/group) or the pro-inflammatory cytokine *Tnf* (control FC = 1.43 ± 0.45, ECS FC = 1.41 ± 0.25, *t*_(28)_ = 0.039, *p* > 0.99, *N* = 6/group).

CCL2 signaling is extensively intertwined with epilepsy, simultaneously serving as a cause *and* consequence of both neuronal hyperexcitability and injury (Bozzi and Caleo, 2016). Indeed, besides the connection to post-SE injury noted above, CCL2 has been implicated in the seizure-promoting effects of systemic inflammation (Cerri et al., 2016), and has also been shown to directly upregulate microglial purinergic signaling (Toyomitsu et al., 2012). Given the surprising ECS-induced increase in CCL2 expression, we next tested for one of the more distinctive facets of the purinergic signaling changes seen in SE-induced microglial activation: the enhancement of microglial responsive motility (Avignone et al., 2008, 2015). Motility of microglial processes across increasing ATP gradients (and toward point sources of extracellular ATP, from diffusion) is an important endogenous response to injury (Davalos et al., 2005), as well as a sensitive in-slice assay of microglial purinergic signaling.

**Figure 5.**
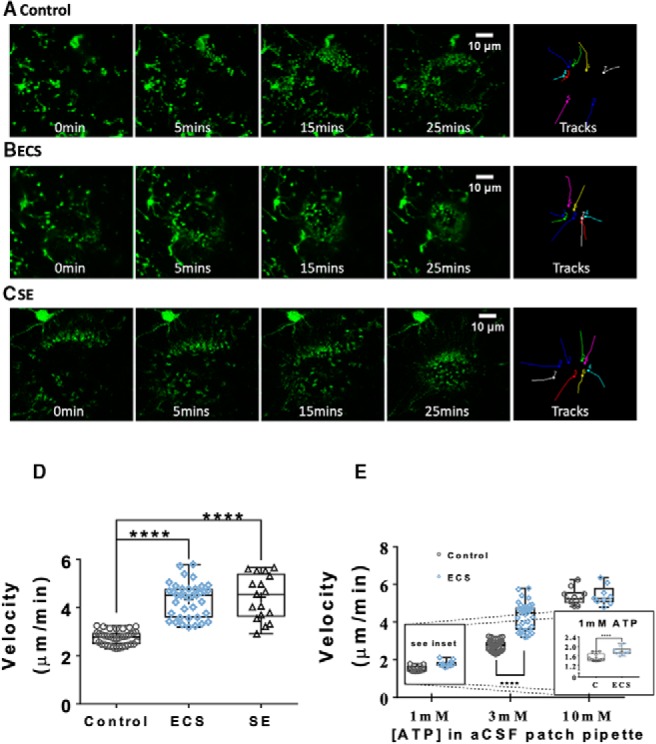
ECS, like SE, potentiated the ATP-responsive motility of microglial processes in acute hippocampal slices. ***A***, ***B***, Representative MIPs of confocal zt-stacks showing the time course of the microglial response (in green) to 3 mM ATP in a patch pipette (in red) in acute hippocampal slices from control (***A***), ECS (***B***), and SE (***C***) animals. ***D***, ECS and SE similarly increased the average process velocity during the microglial response to 3 mM ATP. ***E***, ECS-induced enhancement of microglial responsive motility is concentration dependent: there was a small but significant difference in the responses to 1 mM ATP, while the responses to 10 mM were not significantly different. ****p < 0.0001.

We thus next investigated the effect of ECS on this microglial behavior, by imaging process extension and migration responses to a pipette containing ATP in aCSF (Fig. 5*A*,*B*). As summarized in Figure 5*C*, microglia from control animals slowly mounted a response in the form of a narrowing circle, formed by the leading edge of the processes as they advanced toward the pipette. Importantly, this directional motility was not evoked by 0 mM [ATP]/aCSF-only pipettes, suggesting that the response was purely to ATP and not to the physical presence of the pipette.

In stark contrast to the responses in control slices, slices from ECS and SE animals displayed similarly enhanced microglial process motility toward 3 mM ATP in a patch pipette, 24 h after the seizures. One-way ANOVA revealed a significant effect of treatment group (*F*_(2,91)_ = 17.9, *p* < 10^−15^). Control slices (*n* = 39 slices from *N* = 10 animals) displayed a mean process velocity of 2.7 ± 0.04 μm/min, compared to 4.3 ± 0.12 μm/min for 38 slices from 10 ECS animals, and 4.4 ± 0.23μm/min for 17 slices from five SE animals (by Tukey’s multiple comparison test; C vs ECS: *q*_(91)_ = 15.7, *p* = 0.0000000004; C vs SE: *q*_(91)_ = 13.1, *p* = 0.0000000004; ECS vs SE: *q*_(91)_ = 0.793, *p* = 0.84). ECS enhancement of ATP-directed microglial process motility was relatively much smaller but still statistically significant when the concentration of ATP in the patch pipette solution was 1 mM (1.5 ± 0.05 μm/min for control vs 1.8 ± 0.05 μm/min for ECS, *t*_(18)_ = 3.79, *p* = 0.0013, *t* test, *n* = 10 slices per group, from *N* = 3 animals per group). A difference between groups was not detectable when the pipette contained a saturating concentration of ATP (10 mM; 5.3 ± 0.14 μm/min for control vs 5.4 ± 0.17 μm/min for ECS, *t*_(17)_ = 0.233, *p* = 0.82, *t* test, *n* = 10 and *n* = 9 slices per group, from *N* = 3 animals per group).

Because we did not observe a change in microglial morphology or spontaneous motility, and the effect of ECS on responsive motility was occluded at the highest ATP concentration, our data suggest that ECS increases purinergic responses within microglia, rather than increasing endogenous ATP release activity guiding microglia.

**Figure 6.**
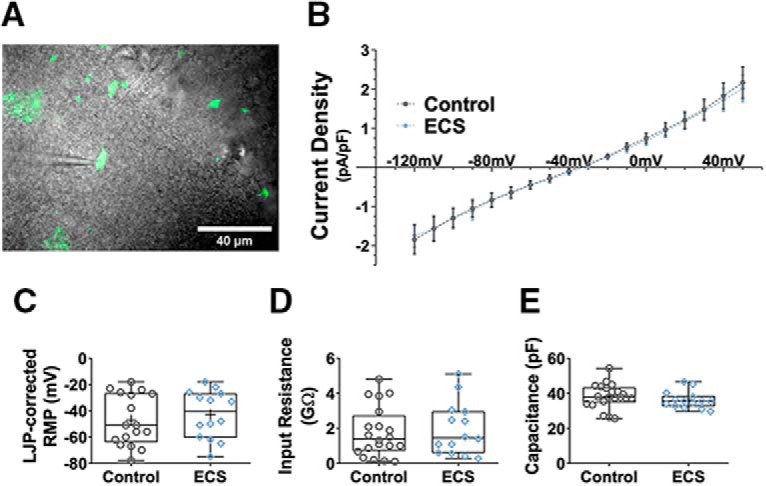
ECS had no effect on intrinsic electrophysiological properties of CA1*sr* microglia. ***A***, Representative photomicrograph showing GFP-labeled microglia superimposed with a 60× DIC image of the hippocampal slice. ***B***, Current density-voltage relation in microglia was unchanged after ECS. Current amplitudes were measured at steady state during 500-ms voltage steps. Data are shown as mean±SEM. ***C–E***, ECS did not affect microglial RMP (**C**), IR (***D***), or membrane capacitance (***E***).

To directly test the hypothesis that ECS enhanced purinergic signaling mechanisms in microglia, and to further characterize hippocampal microglia after ECS, we used patch-clamp electrophysiology targeting fluorescently tagged microglia (Fig. 6) as had previously been done after SE (Avignone et al., 2008). As measured by whole-cell recordings in acute hippocampal slices, control microglia displayed the expected negligible voltage-activated currents, bi-modal distribution of negatively polarized RMPs, high IR, and relatively low capacitance. ECS did not affect the intrinsic electrophysiological properties of CA1*sr* microglia: we did not detect any induction of Kv voltage-activated potassium currents or changes in I/V curves in the voltage range tested (effect of treatment group: *F*_(1468)_ = 0.165, *p* = 0.69, two-way ANOVA, *n* = 16 and *n* = 12 cells; Fig. 6*B*), and the distributions of RMP (*t*_(29)_ = 0.635, *p* = 0.53, *t* test, *n* = 17 and *n* = 14; Fig. 6*C*), IR (*t*_(32)_ = 0.348, *p* = 0.73, *t* test, *n* = 20 and *n* = 14; Fig. 6*D*), and membrane capacitance (*t*_(33)_ = 1.02, *p* = 0.316, *t* test, *n* =21 and *n* = 14; Fig. 6*E*) were all similar across both groups (*N* = 3 and *N* = 4 mice for the control and ECS groups, respectively) and in accordance with published ranges (Avignone et al., 2008; Menteyne et al., 2009; Kettenmann et al., 2011; Verkhratsky and Noda, 2014; de Biase et al., 2017; Madry et al., 2018).

**Figure 7.**
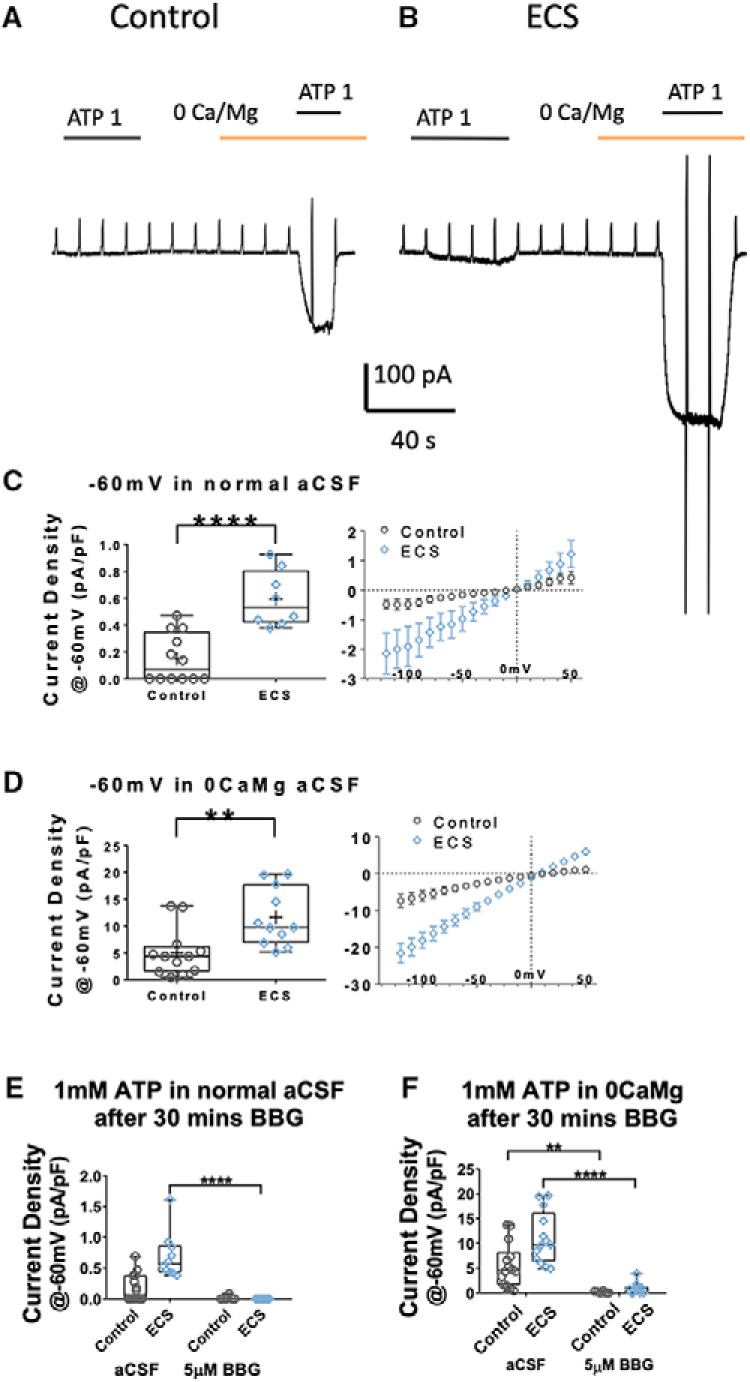
ECS enhanced P2X7 current density in CA1*sr* microglia. ***A***, ***B***, Representative voltage-clamp traces showing the currents induced by local application of 1 mM ATP (black bar) in normal or 0CaMg aCSF (yellow bar), in microglia in slices from sham (***A***) versus ECS (***B***) animals. Cells were held at Vm = −60 mV, with 500 ms ramps from −120 to +50 mV every 10 s. ***C***, ***D***, Peak current density (current amplitude/cell capacitance, pA/pF) of 1 mM ATP-evoked currents in normal (***C***) and divalent cation-free/0CaMg aCSF (***D***) at Vm = −60 mV. ECS resulted in significantly increased current densities under both recording conditions. Panels on the right represent the average ATP-induced current/voltage relation obtained by subtracting the I/V curve obtained before from that obtained during the ATP application. As is expected for P2X-mediated currents, the I/V relation is linear and reverses around 0 mV. ***E***, Peak current density (current amplitude/cell capacitance, pA/pF) of 1 mM ATP-evoked currents in normal aCSF at Vm = −60 mV, with or without preincubation in the specific P2X7 antagonist BBG. BBG significantly reduced the current density evoked by 1 mM ATP in normal aCSF in ECS cells only. ***F***, Peak current density (pA/pF) of 1 mM ATP-evoked currents in divalent cation-free/0CaMg aCSF at Vm = −60 mV, with or without BBG preincubation. BBG significantly reduced the current density evoked by 1 mM ATP in 0CaMg aCSF in both control and ECS cells. **p < 0.01, ****p < 0.0001.

Local perfusion of 1 mM Na-ATP through a Y-tube application device rapidly and reproducibly evoked inward currents in cells from control slices which reversed near 0 mV and were potentiated by divalent cation-free aCSF, putatively identifying them as cationic purinergic currents mediated by P2X receptors (Khakh et al., 2001; Jarvis and Khakh, 2009). ECS significantly increased the current density of the response under both conditions, as illustrated in the example traces in Figure 7*A,B* and summarized in Figure 7*C*,*D*. In normal aCSF, control cells had a mean current density of 0.152 ± 0.052 pA/pF in response to 1 mM ATP when held at Vm = −60 mV, which was significantly smaller than the 0.596 ± 0.073 pA/pF in ECS cells (*t*_(18)_ = 5.07, *p* = 0.000079, *t* test, *n* = 12 and *n* = 8; Fig. 7*C*). In divalent cation-free aCSF, control cells had a mean evoked current density of 5.02 ± 1.28 pA/pF, which was significantly smaller than the 11.6 ± 1.62 pA/pF in ECS cells (*t*_(21)_ = 3.23, *p* = 0.0041, *t* test, *n* = 12 and *n* = 11; Fig. 7*D*).

Next, we sought to identify the receptors underlying these enhanced currents by preincubation of slices with the purinergic inhibitor BBG (5 μM) which preferentially blocks P2X7 over P2X4 and P2X1 (Jiang et al., 2000). Microglia in BBG-treated slices from both control and ECS-treated animals failed to display a current response to 1 mM ATP in normal aCSF (*n* = 6 and 11 cells from *N* = 2 animals/group, Kruskal–Wallis test, *H*_(3)_ = 27.8, *p* = 0.000004; Fig. 7*E*). This was particularly striking in the ECS group, where we detected measurable currents in all cells incubated in normal aCSF and failed to detect any response in cells from slices incubated with BBG. Multiple comparisons tests revealed a significant decrease in current density between ECS cells incubated in aCSF versus those incubated in BBG (Dunn’s test, *p* = 0.000002). Given that the currents evoked in control slices incubated with aCSF were small (and only detectable in half the cell we recorded from), this group did not differ following incubation in BBG (Dunn’s test, *p* = 0.411).

Similarly, as summarized in Figure 7*F*, BBG preincubation drastically reduced the current density elicited by 1 mM ATP in 0CaMg/divalent cation-free aCSF: we recorded mean responses of 0.156 ± 0.073 pA/pF for 6 cells from 2 control animals and 0.92 ± 0.38 pA/pF for 10 cells from 2 ECS animals (Kruskal–Wallis test, *H*_(3)_ = 29.9, *p* = 0.000001). Multiple comparisons tests revealed significant differences in ATP-evoked current densities from: cells from control slices exposed to BBG versus those that were not BBG-treated (Dunn’s test, *p* = 0.0052) and cells from ECS slices exposed to BBG versus those that incubated in control aCSF (Dunn’s test, *p* = 0.00002). These findings are consistent with higher affinity, larger conductance, or increased number of P2X7-containing receptors in hippocampal microglia post-ECS.

**Figure 8.**
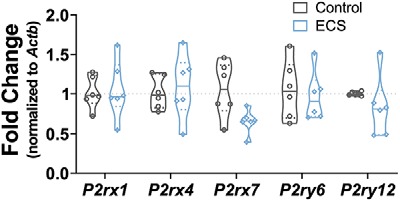
ECS did not change microglial expression of purinergic receptor transcripts. After MACS isolation 24 h after ECS or sham ECS, microglial RNA samples were studied by hydrolysis probe-based qPCR. Relative fold change for each purinergic receptor transcript assayed (the ionotropic receptors *P2rx1*, *P2rx4*, and *P2rx7* and the metabotropic receptors *P2ry6* and *P2ry12*) was determined by the 2^–ΔΔCt^ method, normalizing to *Actb* levels. ECS did not have statistically significant effects on the expression of any of the studied genes.

We next sought to quantify the relative expression of various purinergic receptor genes in microglia after ECS. As illustrated in Figure 8, the above described changes in microglial purinergic function were not accompanied by increases in expression of receptor mRNA, as measured by qPCR: two-way repeated measures mixed model ANOVA analysis showed no effect of treatment group (*F*_(1,25)_ = 2.213, *p* = 0.149), of interaction between treatment group and gene (*F*_(4,25)_ = 1.377, *p* = 0.270) or of gene assayed (*F*_(4,25)_ = 0.8903, *p* = 0.484). There were no statistical differences in relative expression between control and ECS microglial samples (all evaluated by Sidak’s test for multiple comparisons) for: *P2rx1* (control FC = 1.02 ± 0.08, ECS FC = 1.05 ± 0.15, *t*_(25)_ = 0.2205, *p* > 0.99, *N* = 6/group), *P2rx4* (control FC = 1.02 ± 0.08, ECS FC = 1.09 ± 0.16, *t*_(25)_ = 0.4724, *p* > 0.99, *N* = 6/group), *P2rx7* (control FC = 1.05 ± 0.14, ECS FC = 0.66 ± 0.15, *t*_(25)_ = 2.476, *p* = 0.102, *N* = 6/group), *P2ry6* (control FC = 1.05 ± 0.15, ECS FC = 1.06 ± 0.13, *t*_(25)_ = 0.5179, *p* > 0.99, *N* = 6/group), or *P2yr12* (control FC = 1.00 ± 0.01, ECS FC = 0.83 ± 0.16, *t*_(25)_ = 1.026, *p* > 0.99, *N* = 6/group). The lack of changes in transcript levels suggest that translational or posttranslational alterations account for the enhanced P2X7-dependent currents we observed after ECS.

## Discussion

Here, we report a spectrum of changes in hippocampal microglia in response to maximal ECS seizures. This ECS-induced “activation” state features changes that partially overlap with those seen after SE. ECS (unlike SE) did not cause observable differences in hippocampal microglial proliferation/density, morphology, spontaneous motility, or intrinsic electrophysiological properties. On the other hand, similarly as after SE, ECS resulted in increased gene and protein expression of *Ccl2*/CCL2, a chemokine with an established role in neuron-glia-inflammation crosstalk in healthy states and in seizures/epilepsy. Moreover, ECS enhanced ATP-responsive motility and ATP-evoked currents in microglia, in the absence of measurable changes in receptor expression. Thus, brief, non-injurious seizures (ECS) partially recapitulate the activation state seen after prolonged, injurious seizures (SE). This overlap suggests that for a subset of features, seizure activity per se, and not neuronal damage, drives microglial responses.

A potential concern raised regarding evaluation of microglia in acute brain slices is that the slicing process itself may alter microglial state or response. Following others (Avignone et al., 2008), we employed the protective cold sucrose slicing technique and used slices within 5 h or less after slicing. Slices from control and ECS animals deteriorated in a similar fashion, as approximated by the ease of obtaining patch-clamp recordings from ramified GFP^+^ cells deep in the slice and the quality of those recordings. Moreover, our findings with SE mirror those previously reported by others using multiple slice preparation approaches. It does remain possible that either ECS or SE makes neurons more vulnerable to the slicing protocol, meaning that some of our observations could arise from a seizure/slicing interaction.

Commonly employed chemoconvulsant-induced SE models are associated with severe damage in the hippocampus (Turski et al., 1984; Covolan and Mello, 2000), above and beyond the sclerosis associated with the human epilepsies (Löscher, 2011; Becker, 2017). As such, it remains plausible that parts of the SE-induced changes in hippocampal microglia are in fact an acute response to the extensive neuronal injury. Our data seem to support this idea; ECS, which is neither epileptogenic nor damaging induced only a subset of features seen after SE. Thus, the marked proliferation, morphologic simplification, enhancement of baseline motility and changes in intrinsic electrophysiological properties observed in hippocampal microglia after SE but not after ECS may be a response to damage. This would be consistent with the canonical pro-inflammatory role of microglia after injury to the healthy brain (Nimmerjahn et al., 2005). The reported time course and anatomic profile of the microglial response to SE lend additional credence to this correlation, since the changes in proliferation and morphology after SE are localized to areas and time periods where neuronal cell death is expected (Covolan and Mello, 2000; Wyatt-Johnson et al., 2017). Furthermore, SE models relying on systemic or local chemoconvulsant application could additionally be having direct drug effects on the microglia, although single microglia do not express kainate or muscarinic receptors (Hammond et al., 2018, data available online at http://www.microgliasinglecell.com/). This potential confound is mostly mitigated by the finding that different models of SE induction are associated with similar microglial activation (Avignone et al., 2008; Menteyne et al., 2009; Eyo et al., 2014; Arisi et al., 2015; Avignone et al., 2015; Eyo et al., 2016b; Schartz et al., 2016; Tian et al., 2017; Wyatt-Johnson et al., 2017).

On the other hand, our data also show that the microglial responses to ECS and SE have characteristics in common. Both models similarly enhanced the velocity motility of microglial processes in response to a local source of ATP in slices, a sensitive functional assay of P2Y12 receptor signaling in microglia, since this is the receptor underlying the process extensions (Haynes et al., 2006; Ohsawa et al., 2010).

Since the ECS-induced increase in velocity was not detected in response to saturating ATP concentrations, this suggests changes in number or function of purinergic receptors in microglia, as opposed to ambient ATP levels or ATP-induced ATP release from astrocytes. This is notable, given that multiple groups have reported increased ATP levels after brain stimulation (Wu and Phillis, 1978; Wieraszko et al., 1989). Conversely, adenosine, which mediates microglial process retraction (Orr et al., 2009), is also increased in the brain after seizures (Ilie et al., 2012; Lovatt et al., 2012). Interestingly, our whole-cell patch-clamp experiments on microglia in hippocampal slices from ECS-exposed animals also showed a greater P2X current density, chiefly mediated by P2X7-containing receptors. These channels possess biophysical characteristics like calcium permeability and conductance sensitization/pore size increase (Liang et al., 2015) that likely underlie their well-established roles in epilepsy and neuroinflammation (Henshall and Engel, 2015; Amhaoul et al., 2016; Beamer et al., 2017). Since directed motility in response to ATP is the first phase of the microglial response to neuronal injury (Davalos et al., 2005), and P2X7 receptors have a role in the ensuing neuroinflammation, it seems that the seizures in both of our studied models are priming microglia to mount both a faster and stronger response to future insults.

We failed to detect any differences in passive properties [RMP, IR, or membrane capacitance (Cm)] or in voltage-activated currents following ECS. These data are consistent with the lack of an effect on morphology and baseline motility, since IR and Cm directly reflect cell membrane properties, and changes in voltage-activated potassium currents underlie pathogenic microglial-neuron contacts resulting in cell death after SE (Fordyce et al., 2005; Menteyne et al., 2009). Thus, our results point to parallel enhancements in metabotropic and ionotropic purinergic signaling within hippocampal microglia after ECS, in the absence of changes in morphology, baseline motility or intrinsic electrophysiological characteristics. In this way, one can reimagine the complex SE-induced activation state as comprising a particular response to seizures, as well as a parallel response to neuronal injury.

CCL2 (also known as MCP-1) is a canonically pro-inflammatory signaling molecule that has been strongly implicated in the post-SE neuronal injury (Foresti et al., 2009; Arisi et al., 2015; Bozzi and Caleo, 2016; Tian et al., 2017) as well as in the seizure-enhancing effects of systemic inflammation (Cerri et al., 2016). Unexpectedly, given the lack of neuronal damage (and epileptogenesis) after ECS as compared to SE, we detected similarly increased CCL2 protein expression in hippocampal lysates from our ECS-exposed animals. In accordance with this finding, we also observed significantly higher expression of *Ccl2* mRNA in microglia after ECS. This change was not accompanied by increased levels of *Tnf* mRNA, another pro-inflammatory cytokine whose expression is increased post-SE (Avignone et al., 2008). Interestingly, upregulated TNF signaling is thought to underlie the pathogenic losses in blood-brain barrier integrity described after SE (Marchi et al., 2007; Kim et al., 2013; Klement et al., 2018). Although the protein experiments were performed on hippocampal lysates obtained from rapid decapitation without transcardial perfusion, where contamination from blood could cloud our analysis, the qPCR data are derived from MACS-purified microglial samples from perfused animals, strengthening our conclusion.

SE, inflammation, and subsequent injury/epileptogenesis are intricately linked through at least two related signaling pathways: fractalkine and interleukin (IL)-1β. Neuronal-microglial fractalkine signaling, and subsequent astrocytic and neuronal IL-1R activation have been extensively implicated in the pathogenesis mechanism observed after SE (Ravizza and Vezzani, 2006; Ali et al., 2015; Eyo et al., 2016a; Tian et al., 2017), as well as in other rodent models of acquired epilepsy (Plata-Salamán et al., 2000).

Besides complicating the picture as far as the role of CCL2 in neuronal injury post-SE, our finding of increased hippocampal CCL2/microglial *Ccl2* after ECS is of particular interest given the fact that this chemotactic cytokine has been found to directly enhance purinergic signaling in microglia by promoting trafficking of receptors like P2X4 to the microglial plasmalemma (Toyomitsu et al., 2012); whether CCL2 influences trafficking of other receptors such as P2X7 is unknown. However, by some reports, microglial as well as neuronal P2X7 levels are increased in TLE patients and rodent SE models (Jimenez‐Pacheco et al., 2013), while transient inhibition of P2X7 resulted in lasting decreases of post-SE neurodegeneration, gliosis and epileptogenesis (Engel et al., 2012; Jimenez-Pacheco et al., 2016).

Unlike the observed increase in CCL2 protein levels which is correlated with an increase in *Ccl2* mRNA abundance, the increases in purinergic receptor function could not be explained by changes in microglial gene expression. Indeed, we observed no significant differences in microglial expression of *P2rx1*, *P2rx4*, *P2rx7*, *P2ry6*, and *P2ry12* mRNAs post-ECS. Notably, while SE models seem to robustly change the number, morphology and purinergic physiology of hippocampal microglia, the effect of SE on purinergic gene expression has incited controversy in the field and seems to depend not only on timing, strain, age, and model, but also on laboratory. For instance, different groups have reported unchanged (Bosco et al., 2018), decreased (Alves et al., 2017), and increased (Avignone et al., 2008) expression of purinergic receptor gene *P2ry12* in the latent phase of similar models of SE. Likewise, the transcript for *P2rx7* has been reported as both being increased (Avignone et al., 2008; Jimenez-Pacheco et al., 2016) and unaffected (Bosco et al., 2018) post-SE. Thus, the direction and even existence of an SE-induced effect on expression of purinergic receptor genes remains controversial. While a change in microglial expression of *P2rx7* would have been the most parsimonious explanation for the increased P2X7 current density, several possibilities, including biophysical and/or pharmacological changes in the P2X7 channel properties could explain these findings and will have to be further investigated.

Other published work has studied the microglial response after ECS (Jinno and Kosaka, 2008; Jansson et al., 2009), but only found significant changes in microglial density and morphologic/functional activation after chronic ECS stimulation (10–30 seizures). Concordant with our present study, one of these previous studies found that single ECS seizures failed to elicit changes in number or morphology of microglia (Jinno and Kosaka, 2008).

While SE and ECS seizures clearly manifest differently in EEG and behavioral measures, both types of seizures have been shown to recruit the hippocampal formation (Ingvar, 1986; Morinobu et al., 1997; Hsieh et al., 1998; Ji et al., 1998; Scorza et al., 2002; Dyrvig et al., 2012; Sinel’nikova et al., 2013). Since persons with epilepsy normally present with acute seizures as opposed to SE, our acute ECS studies may model human seizures with higher validity than SE or chronic stimulation protocols (by design, these tend to model epileptogenesis rather than acute seizures). It remains unclear for now what the role of microglial changes is in deciding the differential outcomes after the seizures from each model. Since ECS in rodents is a near-perfectly valid model for ECT in humans, our present data may point to a potential role in ECT’s effects, although ECT in the clinic is administered chronically and no mood-stabilizing effects have been shown after single ECT sessions. Further research (including chronic treatments and experiments on mouse models of depression) are needed to elucidate whether the observed microglial response extends to ECT as used in the clinic.

In conclusion, we describe here a state of microglial activation in the mouse hippocampal area CA1*sr* 1 d after a single ECS-evoked seizure. Surprisingly, the observed microglial changes partially overlapped those seen after epileptogenic SE seizures. We posit that the changes present in the response to both models could represent a “signature” of maximal seizures in hippocampal microglia, with little sensitivity to the degree of damage or ensuing epileptogenesis. Repeated ECS in mice is a near-perfect model of ECT in humans: as such, our results also raise a potential role for microglial changes in mediating either ECT’s established benefits (anti-depressant/mood-stabilizing) or its equally well-known deleterious effects (confusion or amnesia).

## Synthesis

Reviewing Editor: Katalin Toth, Universite Laval Decisions are customarily a result of the Reviewing Editor and the peer reviewers coming together and discussing their recommendations until a consensus is reached. When revisions are invited, a fact-based synthesis statement explaining their decision and outlining what is needed to prepare a revision will be listed below. The following reviewer(s) agreed to reveal their identity: Chris Dulla. Note: If this manuscript was transferred from JNeurosci and a decision was made to accept the manuscript without peer review, a brief statement to this effect will instead be what is listed below.

The authors have carefully addressed all the issues raised in the previous review cycle. They provided additional experimental data where appropriate and modified the text where it was required. The manuscript provides high quality data and addresses and important issue.
